# Aβ and tau clearance through aerobic exercise: unveiling the β2-adrenergic receptor’s role in regulating autophagy-lysosomal pathways

**DOI:** 10.1080/27694127.2025.2572512

**Published:** 2025-10-28

**Authors:** Liu Yang, Haitao Yu, Gao-Shang Chai

**Affiliations:** a Department of Fundamental Medicine, Wuxi School of Medicine, Jiangnan University, Wuxi, Jiangsu, China; b MOE Medical Basic Research Innovation Center for Gut Microbiota and Chronic Diseases, Wuxi School of Medicine, Jiangnan University, Wuxi, Jiangsu, China; c Department of Pathology, Affiliated Hospital of Jiangnan University, Wuxi, China

**Keywords:** β2-adrenergic receptor, aerobic exercise, Alzheimer’s disease, amyloid-β, autophagy, tau

## Abstract

The systematic dissection of molecular mechanisms through which aerobic exercise (AE) mitigates neurodegenerative pathologies remains a significant challenge. Alzheimer’s disease (AD) is characterized by impaired autophagy-lysosomal flux and the accumulation of amyloid-β (Aβ) and hyperphosphorylated tau. We recently identified the β2-adrenergic receptor (β2-AR) as a key mediator of exercise-induced bene = d sought to dissect its role in regulating distinct proteostatic pathways. We revealed that AE activates β2-AR signaling to promote lysosomal acidification via upregulation of VMA21, an essential assembly factor for the vacuolar ATPase (V-ATPase) proton pump, thereby facilitating Aβ clearance. Concurrently, AE enhanced autophagosome–lysosome fusion through the β2-AR – retinoid X receptor alpha (RXRα) – charged multivesicular body protein 4B (CHMP4B) axis, promoting tau degradation. Critically, pharmacological inhibition of β2-AR fully abolished these effects. Here, we propose an integrated mechanism through which β2-AR activation by AE could coordinate dual autophagy-lysosomal recovery processes and suggest that targeting this pathway offers a promising therapeutic strategy for AD and related proteostatic disorders.

Alzheimer’s disease (AD), which is characterized by the pathological aggregation of amyloid-β (Aβ) and hyperphosphorylated tau proteins, is closely linked to impairments in the autophagy-lysosomal degradation system. Lysosomal dysfunction, particularly defects in acidification, has been identified as a major factor in AD pathogenesis. Impaired function of the vacuolar ATPase (V-ATPase) proton pump compromises lysosomal acidification, resulting in inefficient clearance of pathological protein aggregates such as Aβ and tau. This disruption of the autophagic flux leads to an accumulation of autophagic vesicles and exacerbates disease progression. Furthermore, autophagy-lysosomal deficits are intimately linked to tau pathology, in which tau accumulation further impedes autolyososomal function, forming a harmful cycle that accelerates neurodegeneration. Compromised proteolytic clearance contributes significantly to the accumulation of toxic protein species, driving neurodegeneration and cognitive decline. Our recent research demonstrates that aerobic exercise (AE) serves as a potent non-pharmacological intervention to mitigate these pathologies through the activation of β2-adrenergic receptor (β2-AR) signaling, which in turn rectifies autophagy-lysosomal flux via distinct molecular mechanisms in Aβ- and tau-driven AD models.

In Aβ pathology, we observed that lysosomal acidification is impaired due to dysfunctional V-ATPase activity, resulting in the accumulation of Aβ within enlarged, hypoacidic autolysosomes. We discovered that AE counteracts this deficit through β2-AR activation, which upregulates vacuolar ATPase assembly factor VMA21 (VMA21), achaperone critical for the assembly of the V-ATPase. Elevated VMA21 ensures the proper organization of V-ATPase subunits, both V₀ and V₁ components, on lysosomal membranes, thereby restoring proton pumping capacity and thus re-acidifying the lysosomal lumen for an efficient enzymatic degradation of Aβ (Figure 1).

**Figure 1.**
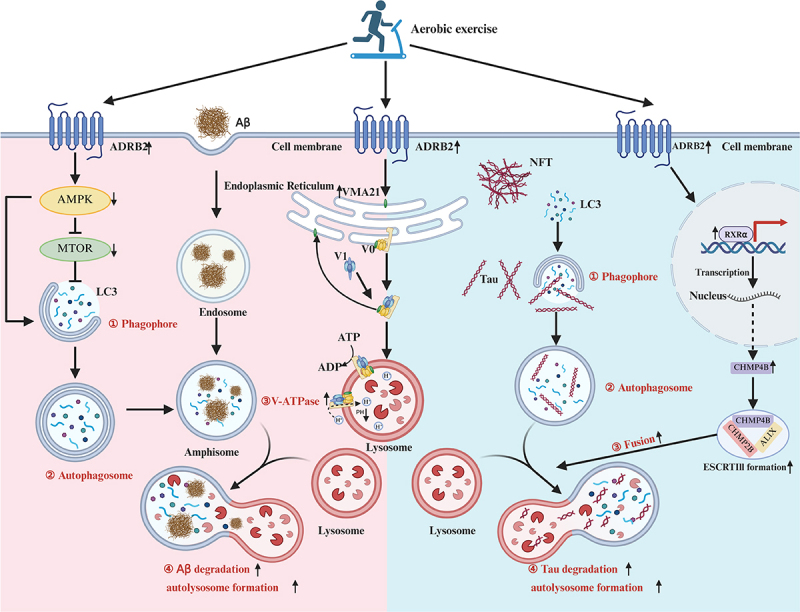
Aerobic exercise activates β2-adrenergic receptors to restore autophagy-lysosomal dysfunction in Alzheimer’s disease. In Aβ pathology (left), β2-AR signaling induces VMA21 expression, promoting V-ATPase assembly on lysosomal membranes and restoring acidification for efficient Aβ degradation. In tau pathology (right), β2-AR activation enhances RXRα-mediated CHMP4B transcription, stabilizing ESCRT-III complexes to facilitate autophagosome–lysosome fusion and tau clearance. This dual mechanism illustrates how exercise targets distinct autophagy defects associated with the Aβ and tau pathologies.

In tau pathology, the accumulation of pathological MAPT/tau suppresses charged multivesicular body protein 4B (CHMP4B) expression, leading to defective assembly of the endosomal sorting complex required for transport III (ESCRT-III) and consequent stalling of autophagosome–lysosome fusion. We demonstrated that AE activates the β2-AR – RXRα (retinoid X receptor alpha) signaling axis, promoting the transcriptional upregulation of CHMP4B. This enhances the stability of ESCRT-III interactions, e.g., with charged multivesicular body protein 2B (CHMP2B) and ALG-2 interacting protein X (ALIX), and facilitates the membrane scission process essential for fusion, thereby restoring autolysosomal degradation of tau aggregates (Figure 1).

A key finding from our studies in APP/PS1 and MAPT P301L transgenic mice is that the benefits of AE are completely abolished by propranolol, a β-adrenergic antagonist, confirming the essential role of β2-AR in mediating exercise-induced autophagy restoration. The convergence of both pathways on β2-AR activation, despite distinct downstream effectors, underscores its role as a central regulatory node in proteostasis restoration. This dual-pathway model illustrates that the therapeutic potential of AE lies in its capacity to broadly target autophagy-lysosomal dysfunction, a common feature in AD progression, rather than being limited to a specific pathology^[1]^.

From a translational perspective, our findings open promising therapeutic avenues. The demonstrated efficacy of β2-AR activation suggests that β2-AR agonists such as terbutaline could replicate the benefits of AE in patients with limited mobility or advanced disease. Moreover, since AD commonly involves both Aβ and tau pathologies, AE’s ability to simultaneously enhance lysosomal acidification and autophagosome–lysosome fusion positions it as a uniquely holistic intervention. Future studies should explore possible synergies between β2-AR activation and existing treatments, such as immunotherapies against Aβ or tau aggregation inhibitors.

Several important research directions emerge from our work. There is a need for systematic quantification of the dose–response relationship between exercise parameters (intensity, frequency, and duration) and the degree of β2-AR-mediated autophagic activation. Such studies could guide the optimization of personalized exercise regiments in clinical and preventive contexts. Furthermore, cell type-specific mechanisms require elucidation; given that β2-AR is expressed across neurons, astrocytes, and microglia, a key future goal is to determine how AE distinctly regulates proteostasis within each of these cellular populations and how their functions are coordinately orchestrated. The temporal aspects of interventions, such as whether AE can reverse preexisting pathology or is most effective in a preventive capacity, should be examined through well-designed longitudinal studies.

In conclusion, our research shows that aerobic exercise represents a powerful, non-pharmacological approach to ameliorating proteostatic dysfunction in AD through the activation of the β2-AR and the subsequent restoration of lysosomal acidification and autophagosome–lysosome fusion. These findings not only shed light on the molecular mechanisms through which lifestyle interventions modulate brain health but also pave the way for novel therapeutic strategies targeting the adrenergic-autophagic pathway in neurodegenerative diseases.

## Data Availability

Data sharing is not applicable to this article as no data were created or analyzed in this study.

## Abbreviations

β2-AR: β2-adrenergic receptor;
AE: aerobic exercise;
ALIX: ALG-2 Interacting Protein X;
AD: Alzheimer disease;
CHMP2B: CHMP2B Charged Multivesicular Body Protein 2B; CHMP4B Charged Multivesicular Body Protein 4B;
ESCRT-III: endosomal sorting complex required for transport III;
RXRα: Retinoid X Receptor alpha;
V-ATPase: vacuolar ATPase;
VMA21: vacuolar ATPase assembly factor VMA21.

## References

[1] Bi S-G, Yu H, Gao T-L, et al. Aerobic exercise attenuates autophagy-lysosomal flux deficits via β2-AR-mediated ESCRT-III subunit CHMP4B in mice with human MAPT P301L. Aging Cell. 2025:e70184. doi: 10.1111/acel.70184 40715737 PMC12507411

